# Improving diagnosis and treatment of knee osteoarthritis in persons with type 2 diabetes: development of a complex intervention

**DOI:** 10.1186/s43058-023-00398-3

**Published:** 2023-02-28

**Authors:** Lauren K. King, Noah M. Ivers, Esther J. Waugh, Crystal MacKay, Ian Stanaitis, Owen Krystia, Jane Stretton, Sim Wong, Alanna Weisman, Zahra Bardai, Susan Ross, Shawn Brady, Marlee Shloush, Tara Stier, Natasha Gakhal, Payal Agarwal, Janet Parsons, Lorraine Lipscombe, Gillian A. Hawker

**Affiliations:** 1 Department of Medicine, University of Toronto, Toronto, ON Canada; 2 Women’s College Research Institute, Women’s College Hospital, Toronto, ON Canada; 3 Department of Community and Family Medicine, University of Toronto, Toronto, ON Canada; 4 Department of Physical Therapy, University of Toronto, Toronto, ON Canada; 5 Patient Research Partner, Toronto, ON Canada; 6 Lunenfeld-Tanenbaum Research Institute, Mount Sinai Hospital, Toronto, ON Canada; 7 Arthritis Rehabilitation and Education Program, Arthritis Society Canada, Toronto, ON Canada; 8 Applied Health Research Centre, Li Ka Shing Knowledge Institute, St. Michael’s Hospital, Unity Health Toronto, Toronto, ON Canada

**Keywords:** Intervention development, Theoretical domains framework, Behaviour change techniques, Osteoarthritis, Type 2 diabetes, Implementation research

## Abstract

**Background:**

Symptomatic knee osteoarthritis (OA) commonly co-occurs in people with type 2 diabetes (T2DM) and increases the risk for diabetes complications, yet uptake of evidence-based treatment is low. We combined theory, stakeholder involvement and existing evidence to develop a multifaceted intervention to improve OA care in persons with T2DM. This was done in partnership with Arthritis Society Canada to leverage the existing infrastructure and provincial funding for community arthritis care.

**Methods:**

Each step was informed by a User Advisory Panel of stakeholder representatives, including persons with lived experience. First, we identified the target groups and behaviours through consulting stakeholders and current literature. Second, we interviewed persons living with T2DM and knee OA (*n* = 18), health professionals (HPs) who treat people with T2DM (*n* = 18) and arthritis therapists (ATs, *n* = 18) to identify the determinants of seeking and engaging in OA care (patients), assessing and treating OA (HPs) and considering T2DM in OA treatment (ATs), using the Theoretical Domains Framework (TDF). We mapped the content to behavioural change techniques (BCTs) to identify the potential intervention components. Third, we conducted stakeholder meetings to ascertain the acceptability and feasibility of intervention components, including content and modes of delivery. Fourth, we selected intervention components informed by prior steps and constructed a programme theory to inform the implementation of the intervention and its evaluation.

**Results:**

We identified the barriers and enablers to target behaviours across a number of TDF domains. All stakeholders identified insufficient access to resources to support OA care in people with T2DM. Core intervention components, incorporating a range of BCTs at the patient, HP and AT level, sought to identify persons with knee OA within T2DM care and refer to Arthritis Society Canada for delivery of evidence-based longitudinal OA management. Diverse stakeholder input throughout development allowed the co-creation of an intervention that appears feasible and acceptable to target users.

**Conclusions:**

We integrated theory, evidence and stakeholder involvement to develop a multifaceted intervention to increase the identification of knee OA in persons with T2DM within diabetes care and improve the uptake and engagement in evidence-based OA management. Our partnership with Arthritis Society Canada supports future spread, scalability and sustainability. We will formally assess the intervention feasibility in a randomized pilot trial.

**Supplementary Information:**

The online version contains supplementary material available at 10.1186/s43058-023-00398-3.

Contributions to the literature

- Given the rising global burden of osteoarthritis (OA), improving the implementation of evidence-based OA care is important.
- This is one of the first studies to address OA care implementation in the context of multimorbidity, focusing on people with type 2 diabetes and OA.
- This complex intervention seeks to improve the provision of evidence-based OA care and reach people with knee OA with and without an existing formal diagnosis.
- User-centred design is important in intervention development to ensure effective outcomes. In addition to describing our systematic approach to intervention development, we outline the involvement of a user advisory panel throughout the research process.

## Background

Driven by the ageing of the population and the epidemic of overweight and obesity, the prevalence of osteoarthritis (OA), the most common form of arthritis, is rapidly rising [1]. This has resulted in an increasing number of people living with OA-related functional limitations and has situated OA as a leading cause of disability worldwide [1]. Knee OA accounts for nearly 80% of the burden of OA [1]. Knee OA-related disability has many potential consequences, including impacts on individuals’ other complex chronic conditions [2–8].

In people with type 2 diabetes (T2DM), OA frequently co-occurs [9] and has detrimental effects [10]. At least one in six individuals with T2DM also has knee OA [11], due to shared risk factors and potentially metabolic pathways [12]. In those with T2DM and knee OA, OA-related walking difficulty increases the risk for diabetes-specific complications and cardiovascular events [4], which may be a result of more sedentary time and/or less engagement in the physical activity [13] that is a cornerstone of T2DM management [14]. Symptomatic OA may also challenge T2DM self-management through poor sleep, low mood and fatigue [15] limiting reserves for the “extra work” of T2DM management [16]. It is therefore incumbent upon the medical community to improve recognition of OA and implementation of evidence-based OA treatment in people with T2DM.

Despite the consequences of knee OA-related functional limitations, safe, effective and guideline-recommended [17] knee OA treatments, such as education, physical activity and weight management, are underused [18]. One problem is the under-diagnosis of OA in the community [19], precluding patient provision of and engagement in care [20, 21]. Those with other chronic conditions, such as T2DM, are even less likely to have their OA addressed [22]. A further challenge is care delivery, with a need for services and programmes to support the necessary behavioural changes that are inherent in OA first-line treatments [23, 24]. Physical activity is also a key treatment for OA, resulting in long-term improvements in pain and function [25]. However, without adequate guidance from health professionals, people often are unclear about what they should do and may avoid participating in physical activity for fear of causing harm [26]. Finally, the current single-condition paradigm for chronic disease management [27] inefficiently slices up care, placing added burden and responsibility on patients for harmonizing chronic disease management strategies. Services that situate OA within the context of multimorbidity may be most successful and best optimize whole-person health.

Multiple complex interventions have been developed in an attempt to put evidence-based OA care into practice, including providing support for the behaviour change required [28]. Most interventions target persons with an established diagnosis of OA and have used strategies such as leveraging non-physician clinicians and/or digital technologies in the provision of care [29–41]. The Goodlife with osteoArthritis in Denmark (GLA:D) programme is an example of a successful education and exercise intervention delivered by trained physical therapists (and other clinicians) to improve pain and function in people with knee and/or hip OA [42]. However, few strategies have been developed to identify, assess and diagnose the many people with joint symptoms consistent with OA who lack a formal OA diagnosis. Marra et al. showed that a complex intervention involving screening persons with knee pain presenting to pharmacies improved the utilization of OA treatments and patient outcomes [43]. To our knowledge, no intervention has been developed specifically to improve OA care in individuals with other complex chronic conditions, such as T2DM, where competing demands may make OA care particularly challenging and necessitate a personalized approach [44]. Overcoming these challenges to improving uptake of and engagement in evidence-based knee OA care in persons with other chronic conditions, such as T2DM, with a view to increasing physical activity, holds the potential to improve both OA outcomes and outcomes related to the other chronic conditions.

Our aim, guided by the UK Medical Research Council (MRC) framework [45], was to use a systematic process combining theory, stakeholder involvement and existing evidence to develop a multifaceted implementation intervention to improve the uptake of evidence-based OA care including physical activity in persons with T2DM and knee OA. A broader aim was to outline this process of systematically developing a complex intervention that seeks to change the behaviours of health professionals (HPs) and patients to provide a template for researchers tackling similar implementation problems.

## Methods

### Setting

In Ontario, Canada, individuals with chronic conditions, such as T2DM, present to primary care providers (family physicians or nurse practitioners) as the first point of contact in the health care system. A referral from a primary care provider or other physician is needed for an individual to access medical specialist services. The health care system in Ontario is publicly funded and privately administered. The Ontario Health Insurance Plan provides coverage for most medical and emergency services provided in Ontario. However, it does not provide universal coverage. Relevant to persons with T2DM and OA, prescription drugs and physiotherapy for those who are not on social assistance and/or under age 65 are paid for out-of-pocket by patients.

### Design

#### Overarching framework

We developed our complex intervention within the first phase of the Treatment of Knee Osteoarthritis in Persons with Diabetes Mellitus (TOP-DM) study, combining relevant theory, current evidence and stakeholder input. The intervention development work took place from 2020 to 2021. We followed the 2008 and 2021 UK MRC updated guidance for the development and evaluation of complex interventions [45, 46] that divide the research process into four phases. As recommended within the intervention development phase, we used theory to comprehensively identify the determinants of behaviour and linked them to the mechanisms of change [47], while meaningfully engaging stakeholders [45], to maximize the potential for developing an intervention that will have positive impacts on health-related outcomes. We also placed strong importance on understanding context throughout the research process, including theorizing how the intervention generates its effects and ensuring it would be implementable among the target population and setting.

#### Arthritis Society Canada

At the conception of this study, we partnered with Arthritis Society Canada, a not-for-profit non-governmental organization in Canada that seeks to elevate arthritis awareness, education and research. Within the province of Ontario, Arthritis Society Canada is directly involved in the provision of arthritis care through the Arthritis Rehabilitation and Education Program (AREP) [48], which provides provincially funded arthritis services, including group and one-on-one education and self-management sessions delivered by a team of trained physical therapists and occupational therapists, at no cost to patients. Thinking ahead to the eventual spread, scalability and sustainability of our intervention, the partnership allowed us to benefit from the existing infrastructure and provincial funding for arthritis care.

#### User advisory panel

We constructed a user advisory panel (UAP) comprising diverse stakeholder membership to facilitate intervention co-design [49–51]. The UAP comprised three patient research partners living with T2DM and OA, the director of AREP and HPs from physical therapy, family medicine, endocrinology and rheumatology. Members of the UAP were consulted throughout the research process.

#### Approach to intervention development

**Fig. 1:**
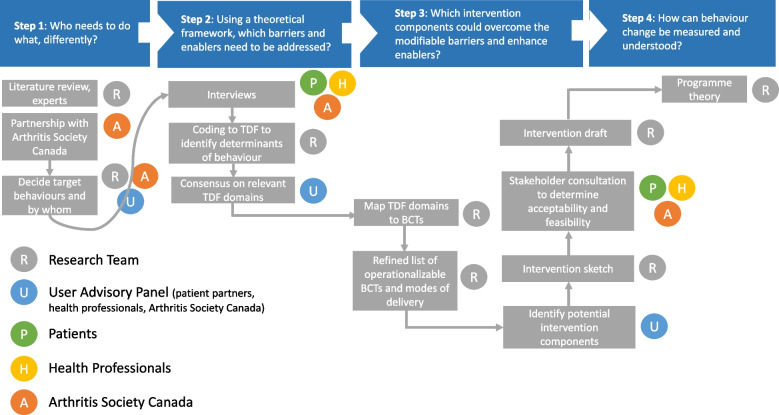
Overview of the step-wise intervention development process. Figure adapted from Riordan et al. [52]. BCT, behavioural change technique; TDF; theoretical domains framework

As the MRC framework lacks detailed operational guidance on the intervention development process, we followed the systematic step-wise approach to intervention development described by French et al. [47]. We outline these four steps below. We also present a summary of our intervention development process in Fig. 1.

Ethics approval was obtained from Women’s College Hospital and University of Toronto Research Ethics Boards.

### Step 1: Who needs to do what, differently?

The process of identifying individuals with T2DM who also have knee OA and providing evidence-based OA care involves multiple separate behaviours being performed by different individuals. Our research team, comprising individuals with expertise in both OA and T2DM, began by brainstorming potential behaviours to target and by whom. We first envisioned the care pathway of a person with T2DM and the steps that are needed for them to have joint symptoms assessed and diagnosed and for evidence-based OA care to be provided. Behaviours were reviewed with and refined through consultation with the UAP.

### Step 2: Using a theoretical framework, which barriers and enablers need to be addressed?

We undertook qualitative studies in three stakeholder groups (patients; diabetes HPs, including family physicians, endocrinologists and diabetes educators; and AREP arthritis therapists [ATs]), to identify the barriers and enablers to the target behaviours [53–55]. Semi-structured telephone interviews, conducted between September 2020 and January 2021, comprehensively explored the behaviours of interest for each group, guided by the Theoretical Domains Framework (TDF) [56]. The interview guides are provided in Additional file 2: Tables B, C and D.

We recruited individuals who had a physician diagnosis of T2DM and knee OA (“patients”), from a hospital-based family medicine clinic and through an email invitation to past clients of AREP. Interviews focused on individuals’ prior experiences living with T2DM and knee OA and behavioural determinants of seeking and engaging in OA care. We purposefully sampled diabetes HPs according to role and practice location to achieve a mix of family physician, endocrinologist and diabetes educator participants and practice locations in Ontario, Canada. Interviews explored the HP experiences with individuals with T2DM who also had knee OA and behavioural determinants of addressing and managing OA. We recruited practising AREP ATs through email invitations. These interviews explored the ATs’ experiences caring for persons with knee OA and other complex chronic conditions and the behavioural determinants of considering T2DM when formulating an OA treatment plan, including prescribing and monitoring physical activity. These interviews also allowed us to better understand the structure and practices within the current AREP care model to enrich our contextual understanding.

All interviews were digitally recorded and transcribed verbatim, and data were organized in NVivo 10. We deductively analysed the data informed by the TDF; within each TDF domain, data were inductively analysed to develop themes/belief statements [57].

### Step 3: Which intervention components (behaviour change techniques and mode(s) of delivery) could overcome the modifiable barriers and enhance the enablers?

#### Mapping TDF domains to appropriate behaviour change techniques

We mapped the barriers and enablers, organized by TDF domains, to behaviour change techniques (BCTs), using the Theory and Technique Tool (https://theoryandtechniquetool.humanbehaviourchange.org/) developed by Michie et al. [58]. A BCT is defined as “a replicable component of an intervention designed to alter or redirect causals processes that regular behaviour” [59]. The tool shows where there are links between BCTs and mechanisms of action (including each TDF domain) based on a literature synthesis and expert consensus. Using this tool, we generated a list of potential BCTs for each identified TDF domain including those with confirmed or inconclusive evidence to support a link.

The list of BCTs was refined by members of the research team as those considered feasible, locally relevant and that could be operationalized within the scope of the current study. Multiple BCTs spanned more than one TDF domain.

#### Developing intervention components and combining them into an acceptable deliverable intervention

To develop intervention components that were likely to be feasible, relevant in the local context and acceptable to stakeholders, we conducted two meetings (2 h each) with our UAP. Meetings were conducted by videoconference and facilitated by two of the authors (LK and GH). At the first meeting, we reviewed the existing literature and our qualitative interview findings and brainstormed potential intervention components. This information was then used by the research team to develop a preliminary sketch of intervention components, considering the APEASE (affordability, practicability, effectiveness, acceptability, side effects, equity) criteria [60]. We drew on practice guidelines for persons with knee OA [17], T2DM [61] and results of prior OA interventions and considered many different potential intervention components and modes of delivery.

At the second meeting, we discussed the sketch of the intervention. We presented unrefined potential components to invite input from our UAP. The UAP deliberated on the modes of delivery of intervention components and how to select and tailor specific strategies to address contextual needs. The research team made revisions to the draft intervention and presented the updates to our three patient partners, separately in 30–60-min meetings, to confirm acceptability and feasibility and whether other alternatives should be considered. Based on these discussions, we made further modifications. We then discussed the proposed intervention with two family physicians from our UAP, one rural and one urban, separately, to review the feasibility of the intervention components in their clinical practices. We reviewed the intervention with a rheumatologist, to confirm the acceptability of the identified ways to address OA. We conducted a meeting with a group of four endocrinologists who practised in different clinical settings to get diverse perspectives on how the intervention could be applied. We then presented and discussed the intervention with stakeholders at Arthritis Society Canada, including three ATs, the director of AREP and the vice president of AREP for Arthritis Society Canada. Some components that were not considered feasible were removed.

### Step 4: How can behaviour change be measured and understood?

We conducted evaluability assessments [45] through engaging experts in quality and innovation (NG) and implementation science (NI) to decide on proximal and feasibility outcomes of the intervention, the data to be collected and assessed and the options for evaluation. This resulted in a plan for feasibility evaluation that will be fully reported separately.

To describe our programme theory, we developed logic models of the final intervention, presenting the inputs, processes and the causal mechanisms by which we expect intervention components to have positive effects.

## Results

The final intervention has been reported according to TiDierR [62].

### Step 1: Identify who needs to do what, differently

We confirmed the following behaviours of interest: (1) for HPs, to identify and treat knee OA; (2) for persons with T2DM and knee OA, to seek and engage in knee OA care; and (3) for Arthritis Society Canada ATs, to consider T2DM when formulating an OA treatment plan, including a focus on prescribing and monitoring physical activity. Using the Action, Actor, Context, Target, Time (AACTT) framework [63], we further specify these behaviours in Table 1.

**Table 1:** Behaviours of interest specified using the Action, Actor, Context, Target, Time (AACTT) framework. Behaviour 1 (persons with type 2 diabetes (T2DM) and knee osteoarthritis (OA)): to seek and engage in knee OA care. Behaviour 2 (health professionals who treat T2DM): to identify and treat knee OA. Behaviour 3 (Arthritis Society Canada arthritis therapists): to consider T2DM when formulating an OA treatment plan, including a focus on prescribing and monitoring physical activity

	**Behaviour 1**	**Behaviour 2**	**Behaviour 3**
**Action**	Present to health professional for OA evaluation and care and engage with OA management	Assess for joint symptoms and treat/refer for treatment if present	Emphasize physical activity for the treatment of OA in the context of T2DM
**Actor**	People with T2DM	Diabetes health professionals (primary care providers, endocrinologists providing diabetes care, diabetes educators)	Arthritis therapists
**Context**	Daily life	Primary care or diabetes clinic	Arthritis care visit
**Target**	People with T2DM and OA	People with T2DM and OA	People with T2DM and OA
**Time**	When symptoms present	When patients attend	When patients attend

### Step 2: Identify the barriers and enablers that need to be addressed using a theoretical framework

We conducted qualitative interviews with 18 persons with T2DM and knee OA, 18 HPs who treat persons with T2DM (8 endocrinologists, 7 family physicians, 3 diabetes educators) and 18 ATs. These studies, reported elsewhere [53–55], are summarized below, and we list the TDF domains that we identified as relevant in parentheses.

#### Interviews with persons living with knee OA and T2DM

Of the 14 TDF domains, seven prominently influenced the behaviour of patients to seek and engage in OA care. Important barriers included the insufficient provision of OA knowledge to fully engage in care (knowledge), feeling incapable of participating in physical activity/exercise due to joint pain (beliefs about capabilities), lack of guidance from HPs and insufficient access to community programmes/supports (environmental context and resources) and being uncertain that OA therapies would help them (optimism). Key enablers were strong social support (social influences), sources of accountability (behavioural regulation) and experiencing benefit from prior use of treatment (reinforcement).

#### Interviews with T2DM health professionals

We identified six TDF domains that prominently influenced the behaviours of HPs to assess and treat knee OA. For all HPs, important barriers included not seeing joint pain as a priority (intention), perceived lack of programmes to which they could refer their patients (environmental context and resources), insufficient knowledge and skills to assess OA, particularly for endocrinologists and diabetes educators (knowledge, skills), belief that it was not within their professional role to address OA (professional role and identity) and that other physicians would not want to receive a referral for OA care (social influences).

#### Interviews with AREP arthritis therapists

We identified five TDF domains that were relevant to the ATs’ behaviour to consider T2DM when formulating a knee OA management plan. ATs’ perceived lack of specific knowledge around comorbidities including T2DM (knowledge); there was a lack of breadth in skills in behavioural change techniques to help patients set and reach their goals, particularly when it came to physical activity (skills); therapists generally had no intention for a patient’s comorbidity profile to influence their treatment recommendations (intention); they saw their role as joint focused (professional role and identity); and lack of a formalized follow-up structure of the current Arthritis Society Canada AREP programme limited sufficient patient monitoring and follow-up (environmental context and resources).

### Step 3: Which intervention components (behaviour change techniques and mode(s) of delivery) could overcome the modifiable barriers and enhance the enablers?

#### Identify potential behavioural change techniques and modes of delivery to overcome barriers and enhance the enablers

Our initial list of BCTs, at each of the patient; HP; and AT levels, is shown in Additional file 1: Table A.

#### Identify what is likely to be feasible, locally relevant and acceptable and combine identified components into an acceptable intervention that can be delivered

At our first UAP meeting, there was a broad agreement with qualitative findings and support for leveraging the Arthritis Society Canada AREP programme infrastructure as a vehicle to provide OA care. UAP members suggested the following ideas to operationalize BCTs and optimize modes of delivery: development of simple ways T2DM clinicians could screen for OA, improving diabetes HPs awareness around the impact of OA, different ways to provide T2DM patients with guidance about exercise for OA and use of diabetes flow sheets to prompt discussion about reasons for physical inactivity, including inquiring about OA. Based on this discussion, we refined our list of BCTs and excluded those deemed outside the scope of the study or not feasible. Our selected operationalizable BCTs, within each domain and mode of delivery, targeting patient, diabetes HP and AT level, are summarized in Additional file 1: Table A.

At the second UAP meeting, all members supported our intervention sketch. In particular, stakeholders from Arthritis Society Canada supported adapting the existing AREP model to deliver longitudinal OA care. There was a widespread interest in ensuring that access to the intervention would be equitable for all and not rely on the need for advanced technology, and therefore, we removed some elements of the proposed intervention that centred around digital technologies. There was however interest in ensuring flexibility in how care was delivered to take into account patient preferences and so designed the intervention to be delivered in-person or virtually (telephone and/or video visits).

There were two main steps of the draft intervention. The first step involved screening for and identification of symptomatic knee OA within diabetes care, with referral to Arthritis Society Canada AREP in those identified as having suspected or confirmed OA for further evaluation and care. The second step involved a longitudinal treatment programme over 4 months delivered by AREP ATs, comprising one-on-one individualized OA management within the context of T2DM and including a focus on supporting the behaviour change requirement to increasing aerobic physical activity. We named this the Arthritis Society Diabetes & Osteoarthritis Program.

During small group meetings, reviewing detailed intervention components, patient partners described that an early check-in would help to support engagement with OA care through promoting accountability and allowing early troubleshooting to take place if any barriers arose. Several physicians emphasized the need to provide communication from the OA programme back to primary care and endocrinology so that care plans could be recognized and reinforced at those clinical encounters. We refined the intervention to incorporate these suggestions. We heard from AREP ATs about specific elements that would be required to support their delivery of OA care as part of the intervention, to prepare them for assessing and treating persons with T2DM and knee OA. We confirmed topics to be delivered in a 1-day workshop for ATs, drawn from results of the AT qualitative interviews, which included an overview of T2DM, behavioural change techniques and health coaching, wearables and technology that can be offered to support patients to meet physical activity goals and an update on the management of knee OA.

**Fig. 2:**
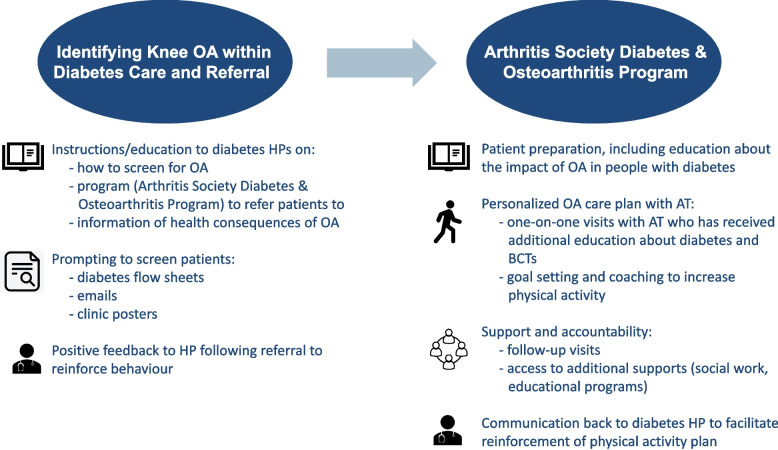
Organization of final intervention, with major components summarized. AT, arthritis therapist; BCT, behavioural change technique; HP, health professional

In Table 2, we show the final intervention components, including content and modes of delivery, mapped to the selected BCT and TDF domain being targeted. We organize this by group (patient, HP and AT); however, the order is not meant to convey the temporality or importance of a single group or behaviour. Intervention steps and major components are shown in Fig. 2.

**Table 2:** Final intervention components mapped to the behavioural change technique (BCT) and theoretical domains framework (TDF) barrier or enabler being targeted, by participant group: (A) patients, (B) health professionals and (C) arthritis therapists

Step 2: Using a theoretical framework, which barriers and enablers need to be addressed?		Step 3: Which intervention components could overcome the modifiable barriers and enhance the enablers?	
**Barrier or enabler**	**TDF domain**	**Behavioural change technique**	**Modes of delivery and content**
**A. Patient**			
Understanding about OA and its management	Knowledge	Instructions on how to perform behaviour	Personalized OA treatment plan
		Information about health consequences	Written information and education through one-on-one AT visits about the interaction between OA and T2DM and the consequences of untreated symptomatic OA
Capability to engage in exercise with joint pain	Beliefs about capabilities	Problem solving	ATs help deconstruct physical activity barriers and co-develop goals
		Instructions on how to perform behaviour	Written and verbal advice on engaging in OA care
		Graded tasks	Individualized goal setting and titration of physical activity
Expecting OA treatment will help	Optimism	Review outcome goal(s)	Personalized OA care plan, including physical activity goals
Feedback (internal or external) that OA treatment is helping	Reinforcement	Feedback on behaviour	AT to develop a monitoring plan with patient for OA care, including PA
		Prompts/cues	AT to develop individualized reminder plan (emails, use of wearable device)
Support from health professionals	Environmental context and resources	Prompts/cues	–
		Social support (practical)	ATs to help the patient determine community supports to meet goals, in addition to communicating care plan to the health care team
		Restructuring of physical environment	Longitudinal relationship with AT to support necessary behavioural change
Access to facilities, programmes and resources	Environmental context and resources	Prompts/cues	–
		Social support (practical)	–
		Restructuring of the physical environment	Diabetes & Osteoarthritis Program provides comprehensive OA care at no out-of-pocket costs
Social support to encourage engagement in OA treatment	Social influences	Social support (practical)	ATs to help connect patients with potential sources of support at home and in their community, including access to Arthritis Society Canada social workers as needed
Peer influence on OA therapies	Social influences	Social support (practical)	Welcome package for Diabetes & Osteoarthritis Program to include peer stories and experiences
Sources of accountability	Behavioural regulation	Goal setting	ATs to deploy a wide range of BCTs to help patients set, titrate, troubleshoot their goals and progress and provide sources of accountability
		Graded tasks	
		Problem solving	
		Prompts/cues	
**B. Health professional**			
Knowledge about OA diagnosis and treatment	Knowledge	Instructions on how to perform behaviour	Electronic educational materials providing information on how to screen for knee OA, including patient screening questions
		Information about health consequences	Electronic educational materials describing health consequences of untreated symptomatic knee OA
Skills in joint examination	Skills	Instructions on how to perform behaviour	Electronic education materials providing screening questions for knee OA that remove the need for physical exam
		Information about health consequences	–
Role in OA management	Social and professional role and identity	Credible source	Educational materials from the study team that include input from endocrinologists and family physicians
Priority of OA care	Intentions	Information about health consequences	Electronic educational materials describing health consequences of untreated symptomatic knee OA
		Information about others’ approval	Each referral to Diabetes & Osteoarthritis Program accompanied by a confirmation note to referring provider with approval and appreciation for referral
Resources for OA care, including access to physical therapy	Environmental context and resources	Prompts/cues	Use of various methods to remind clinicians of Diabetes & Osteoarthritis Program (modification of diabetes flow sheets, clinic posters)
		Conserving mental resources	Efficient referral process with little time of referring provider required
		Restructuring the physical environment	Creation of Diabetes & Osteoarthritis Program as a resource to refer to for timely OA care at no cost to the patient
Perception rheumatologists do not want to manage OA	Social influences	Information about others’ approval	Each referral to Diabetes & Osteoarthritis Program accompanied by a confirmation note to referring provider with approval and appreciation for referral
**C. Arthritis therapist**			
Paucity of specific diabetes knowledge	Knowledge	Instructions on how to perform behaviour	Workshop for ATs to increase T2DM-specific knowledge
		Information about health consequences	
Variability in skills in behaviour change techniques	Skills	Instructions on how to perform behaviour	Workshop for ATs to increase confidence in the use of BCTs
		Information about health consequences	
Variability in perceived role in optimizing overall health	Social and professional role and identity	Credible source	Arthritis Society Canada leadership supporting a focus on whole-person health
		Social support	
Variable intention to consider comorbidity in OA management plan	Intention	Information about health consequences	Workshop for ATs to increase knowledge about the impact of OA on other chronic conditions, including T2DM and importance of physical activity
		Information about others’ approval	Arthritis Society Canada leadership supporting a focus on whole-person health
		Goal setting	
Existing AREP programme limits the provision of longitudinal OA care	Environmental context and resources	Prompts/cues	Physical goals and plan sheet for ATs to complete with patients
		Restructuring of the physical environment	Through the creation of Diabetes & Osteoarthritis Program, follow-up visits scheduled over 6 months

“–” indicates not applicable

### Step 4: How can behaviour change be measured and understood?

#### Programme theory

**Fig. 3:**
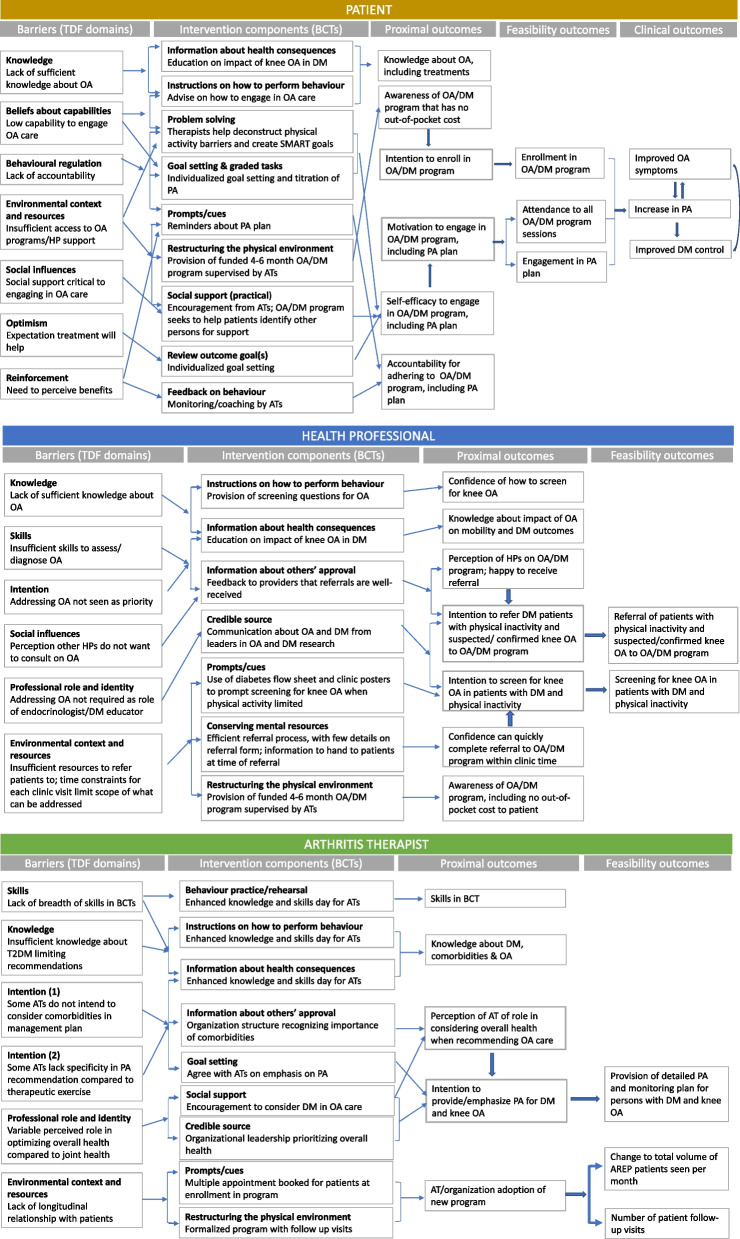
Logic models of the multi-level intervention for **A** patients, **B** health professionals and **C** arthritis therapists. Barriers and enablers according to the Theoretical Domains Framework (TDF) are mapped to behavioural change techniques and then proximal, feasibility and clinical outcomes. AT, Arthritis Therapist; BCT, behavioural change technique; HP, health professional; PA, physical activity; TDF; Theoretical Domains Framework

We expect our intervention to work by enabling change in the behaviours of patients, diabetes HPs and ATs, as shown in our logic models (Fig. 3). For patients, our intervention will increase intention and motivation to engage in OA care, through both facilitating receipt of a diagnosis of OA and providing support for management. For HPs, it will increase the intention to screen for knee OA and refer for assessment and treatment when OA is suspected or confirmed. For ATs, through adapting the existing AREP programme and creating the Arthritis Society Diabetes & Osteoarthritis Program, we have created an environmental change to support the provision of individualized longitudinal care. This programme also shifts the focus in care from joint-specific therapeutic exercise to increase overall physical activity. We intend for there to be flexibility in the delivery of intervention components to allow for variation in practices of different diabetes HPs, including clinic resources, yet maintain the integrity of the core intervention components [45].

## Discussion

In this paper, we describe the development phase of a multifaceted intervention to overcome barriers to the assessment and diagnosis of OA in persons with T2DM. The TOP-DM intervention promotes evidence-based OA treatment with a focus on physical activity, including the mechanisms to support the behaviour change this requires, given its importance in both T2DM and OA care. We expect our intervention to work by enabling change in behaviours of patients, diabetes HPs and ATs and have targeted multiple groups given the complexity of this health challenge. In keeping with MRC guidance [45], our intervention development process has incorporated theory, in this case, of behaviour change [64], existing evidence and stakeholder involvement, while considering local context, to maximize the likelihood of success. The final intervention brings together a range of components that were specifically developed in the context of concomitant T2DM. Some of the components are similar to those incorporated in prior knee OA interventions, including strategies to screen for knee OA [43], increase health professional knowledge [65, 66], provide patient education [67, 68] and improve uptake of physical activity through health professional support [42].

We involved multiple stakeholders in a co-design process, to develop our intervention alongside those for whom it is designed [49–51]. Our UAP brought together patient partners, Arthritis Society Canada and diverse HPs, in a focus group-like setting where concepts could be tackled from many important perspectives. Given the focus on implementation in the context of multiple chronic conditions, this involved a large number of individuals. One lesson learned was that when bringing a large group together, any one individual could get relatively little “air time”. To address this, we also engaged stakeholders (patients, Arthritis Society Canada and HPs) individually or in small stakeholder groups to allow sufficient time to garner their inputs and to mitigate any possible hierarchal dynamics that might prevent individuals from expressing their views. Teams undertaking implementation research should carefully consider the modes in which they plan to engage stakeholders [69].

Strengths of this work include the use of a systematic step-wise approach [47]. Through the use of theory, and linking identified barriers to health behaviour to relevant and effective BCTs, we have explicitly outlined how we expect our intervention to work, and we will be able to evaluate these proposed mechanisms of change in future work. With our transdisciplinary approach, including collaborating with end-users and community stakeholders throughout the research process, we have sought to enhance the potential feasibility and effectiveness of our intervention [70, 71]. This work fills an important gap. To our knowledge, our intervention is one of only a few seeking to increase the identification and diagnosis of individuals with symptomatic knee OA to facilitate care, and none to our knowledge has done so within the context of another complex chronic condition. Our work to integrate OA care within T2DM complex chronic disease management is in an effort to break down the current, mostly siloed, models of chronic disease care. Our intervention leverages the existing Arthritis Society Canada AREP infrastructure and provincial funding, supporting potential intervention spread, scalability and sustainability.

Our work has some limitations. First, this approach to intervention development requires significant time and resources. While explicit use of theory has several advantages, including helping to inform important intervention elements [72], the evidence base to support that theory-informed interventions are superior to those not based on theory is sparse, largely due to the challenges of empirically addressing this question [73]. Multiple theories and frameworks of individual and organizational behaviour change exist, with little consensus on how to optimally select one [74]. We selected the TDF as it is recognized as the most comprehensive framework for designing implementation interventions [47]; however, other frameworks or theories can be used. While we sought to bridge the distance between OA and T2DM care, we expect many patients to have additional chronic conditions that may present additional barriers to OA care that were not explicitly addressed through this intervention. Our intervention may have limited generalizability given the use of AREP, as other jurisdictions may not have a similar infrastructure.

## Conclusions

In conclusion, using a systematic process combining theory, stakeholder involvement and existing evidence, we have developed a complex implementation intervention to improve OA care in persons with T2DM with the goal to improve both OA and T2DM outcomes and optimize overall health and well-being. While we have used robust methods in development, our next steps include assessment of proximal and feasibility outcomes using rapid-cycle change quality improvement methods and engaging potential intervention users to inform refinements to our intervention before evaluation of both feasibility and effectiveness outcomes in a pilot cluster randomized clinical trial.

## Supplementary Information

**Additional file 1: Table A.** Our initial list of behavioural change techniques (BCTs), mapped to each relevant theoretical domains framework (TDF) domain for A) Patients, B) Health professionals, and C) Arthritis therapists with either a confirmed link or inconclusive evidence for a link according to the Theory and Technique Tool (https://theoryandtechniquetool.humanbehaviourchange.org/) (70). Those in bold font indicates the BCTs selected in the research process as potentially operationalizable and feasible.

**Additional file 2: Table B.** Interview Guide: Patients with Diabetes and Osteoarthritis. **Table C.** Interview Guide: Physicians and Diabetes Educators. **Table D.** Interview Guide: Arthritis Therapists.

## Data Availability

Data sharing is not applicable to this article as no datasets were generated or analysed during the current study.

## Abbreviations

AACTT: Action, Actor, Context, Target, Time framework
APEASE: Acceptability, Practicability, Effectiveness, Affordability, Side-effects, and Equity criteria
AREP: Arthritis Rehabilitation and Education Program
AT: Arthritis therapist
BCT: Behavioural change technique
GLA:D: GoodLife with osteoArthritis in Denmark
HP: Health professional
MRC: UK Medical Research Council
OA: Osteoarthritis
T2DM: Type 2 diabetes
TiDieR: Template for Intervention Description and Replication Checklist
TDF: Theoretical Domains Framework
TOP-DM: Treatment of Knee Osteoarthritis in Persons with Diabetes Mellitus
UAP: User advisory panel
