# Supplementary material for: Improving diagnosis and treatment of knee osteoarthritis in persons with type 2 diabetes: development of a complex intervention
Source: Implement Sci Commun. 2023 Feb 28;4:20. doi: 10.1186/s43058-023-00398-3 (PMC9972628; doi:10.1186/s43058-023-00398-3)
Supplement: Supplementary file 1 — Additional file 1: Table A. Our initial list of behavioural change techniques (BCTs), mapped to each relevant theoretical domains framework (TDF) domain for A) Patients, B) Health professionals, and C) Arthritis therapists with either a confirmed link or inconclusive evidence for a link according to the Theory and Technique Tool (https://theoryandtechniquetool.humanbehaviourchange.org/) (70). Those in bold font indicates the BCTs selected in the research process as potentially operationalizable and feasible. [file 43058_2023_398_MOESM1_ESM.docx]

**Supplementary File A**

Our initial list of behavioural change techniques (BCTs), mapped to each relevant theoretical domains framework (TDF) domain for A) Patients, B) Health professionals, and C) Arthritis therapists with either a confirmed link or inconclusive evidence for a link according to the Theory and Technique Tool ([*https://theoryandtechniquetool.humanbehaviourchange.org/*](https://theoryandtechniquetool.humanbehaviourchange.org/))(70). Those in bold font indicates the BCTs selected in the research process as potentially operationalizable and feasible.

1. **PATIENTS**

| **Using a theoretical framework, which barriers and enablers need to be addressed? (Step 2)** | **Within which theoretical domains do the barriers and enablers operate?** | **Which intervention components (behaviour change techniques and mode(s) of delivery) could overcome the modifiable barriers and enhance the enablers? (Step 3)*** |
| --- | --- | --- |
| Lack of sufficient knowledge about OA to understand rationale to treat, to engage in physical activity | Knowledge | - Biofeedback - **Instructions on how to perform behaviour** - **Information about antecedents** - **Information about health consequences** - **Information about social and environmental consequences of performing behaviour** - Feedback on behaviour |
| Beliefs about capabilities relating to engaging in physical activity in the context of having joint pain | Beliefs about capabilities | - **Problem solving** - **Instruction on how to perform behaviour** - Demonstration of behaviour - Behavioural practice/rehearsal - **Graded tasks** - **Verbal persuasion about capability** - **Focus on past success** - Self-talk - **Goal setting (behaviour)** - Biofeedback - **Social reward** - **Reduce negative emotions** |
| Insufficient optimism that treatment will be successful | Optimism | - **Review outcome goals** |
| More likely to engage with OA treatments when have experienced benefits | Reinforcement | - Material incentive (behaviour) - Material reward (behaviour) - Non-specific reward - **Social reward** - Non-specific incentive - Inventive (outcome) - Reward (outcome) - Punishment - **Monitoring of behavior by others without feedback** - **Feedback on behaviour** - **Prompts/cues** - Associative learning - **Self-reward** |
| Prior negative experiences with health care providers | Environmental context and resources | - **Social support (practical)** - **Prompts/cues** - Remove adverse stimulus - Restructuring physical environment - Avoidance/reducing cues for the behaviour - **Conserving mental resources** - Problem solving |
| Insufficient access resources to refer to and/or support patients with osteoarthritis | Environmental context and resources | - **Social support (practical)** - **Prompts/cues** - Remove adverse stimulus - **Restructuring physical environment** - Avoidance/reducing cues for the behaviour - **Conserving mental resources** - Problem solving |
| Social support as crucial enabler to engaging in OA treatment | Social influences | - **Social support (unspecified)** - **Social support (practical)** - Social comparison - **Information about others’ approval** - Social reward - **Restructuring social environment** - **Monitoring of behaviour by others without feedback** |
| Accountability to stay engaged with my OA treatments crucial | Behavioural regulation | - **Problem solving** - **Self-monitoring of behaviour** - Information about antecedents - Behaviour substitution - Reduce negative emotions - **Conserving mental resources** - **Goal setting (behaviour)** - **Action planning** - **Discrepancy between current behaviour and goal** - **Behavioural contract** - Self-monitoring of outcomes of behaviour - Habit formation - Habit reversal - Punishment |

*BCTs with supportive or inconclusive evidence related to each TDF domain

1. **HEALTH PROFESSIONALS**

| **Using a theoretical framework, which barriers and enablers need to be addressed? (Step 2)** | **Within which theoretical domains do the barriers and enablers operate?** | **Which intervention components (behaviour change techniques and mode(s) of delivery) could overcome the modifiable barriers and enhance the enablers? (Step 3)*** |
| --- | --- | --- |
| Lack of sufficient knowledge about OA to make a diagnosis and/or recommend treatment | Knowledge | - Biofeedback - **Instructions on how to perform behaviour** - **Information about antecedents** - **Information about health consequences** - **Information about social and environmental consequences of performing behaviour** - Feedback on behaviour |
| Lack of skill to perform joint examination (Endo/DM educator) | Skills | - **Instructions on how to perform behaviour** - Behavioural practice/rehearsal - Graded tasks - Problem solving - Demonstration of the behaviour - Generalization of target behaviour - Self-reward |
| Osteoarthritis is not seen as a comorbidity that should be evaluated by endocrinologist/diabetes educator (Endo/DM educator) | Professional role and identity | - **Social support (unspecified)** - Social comparison - **Credible source** - Identify associated with changed behaviour |
| Addressing joint pain is not seen as a priority | Intentions | - Goal setting (behaviour) - **Information about health consequences** - Incentive (outcome) - **Information about others’ approval** - Valued self-identity |
| Insufficient resources to refer to and/or support patients with osteoarthritis | Environmental context and resources | - **Social support (practical)** - **Prompts/cues** - Remove adverse stimulus - Restructuring physical environment - Avoidance/reducing cues for the behaviour - **Conserving mental resources** - Problem solving |
| Perception that other specialists do not want to receive consultations for osteoarthritis | Social influences | - **Social support (unspecified)** - Social support (practical) - Social comparison - **Information about others’ approval** - Social reward - Restructuring social environment - Monitoring of behaviour by others without feedback |

*BCTs with supportive or inconclusive evidence related to each TDF domain

1. **ARTHRITIS THERAPISTS**

| **Using a theoretical framework, which barriers and enablers need to be addressed? (Step 2)** | **Within which theoretical domains do the barriers and enablers operate?** | **Which intervention components (behaviour change techniques and mode(s) of delivery) could overcome the modifiable barriers and enhance the enablers? (Step 3)*** |
| --- | --- | --- |
| **Lack of specific knowledge limits greater consideration of T2DM and other comorbidities** | Knowledge | - Biofeedback - **Instructions on how to perform behaviour** - **Information about antecedents** - **Information about health consequences** - **Information about social and environmental consequences of performing behaviour** - Feedback on behaviour |
| **Lack of breadth of skills in behavioural change techniques to sufficiently tackle the added challenge of comorbidity** | Skills | - **Instructions on how to perform behaviour** - **Behavioural practice/rehearsal** - Graded tasks - Problem solving - **Demonstration of the behaviour** - Generalization of target behaviour - Self-reward |
| **Perceived role focused on joint health** | Social & professional role and identity | - **Social support (unspecified)** - Social comparison - **Credible source** - Identify associated with changed behaviour |
| **Variable intention to factor comorbidity into OA management plan** | **Intention** | - Goal setting (behaviour) - **Information about health consequences** - Incentive (outcome) - **Information about others’ approval** - Valued self-identity |
| **Existing AREP program limits provision of longitudinal OA care** | **Environmental context and resources** | - **Social support (practical)** - **Prompts/cues** - Remove adverse stimulus - **Restructuring physical environment** - Avoidance/reducing cues for the behaviour - **Conserving mental resources** - Problem solving |

*BCTs with supportive or inconclusive evidence related to each TDF domain
