# Supplementary material for: Improving diagnosis and treatment of knee osteoarthritis in persons with type 2 diabetes: development of a complex intervention
Source: Implement Sci Commun. 2023 Feb 28;4:20. doi: 10.1186/s43058-023-00398-3 (PMC9972628; doi:10.1186/s43058-023-00398-3)
Supplement: Supplementary file 2 — Additional file 2: Table B. Interview Guide: Patients with Diabetes and Osteoarthritis. Table C. Interview Guide: Physicians and Diabetes Educators. Table D. Interview Guide: Arthritis Therapists. [file 43058_2023_398_MOESM2_ESM.docx]

**Supplementary Table B**

**Interview Guide: Patients with Diabetes and Osteoarthritis**

*Thank you for agreeing to speak with me today. I’m going to begin the interview by asking you a few questions in order to get to know you and to learn about your experience with having diabetes and osteoarthritis. We are going to be doing a study that tests an intervention to improve treatment of joint pain in people with diabetes but before we start we really want to hear from people like yourself who are living with both of these conditions. These questions serve as a guide only. We encourage you to talk about any aspect of the topic you wish. There are no right or wrong answers to these questions. Questions will start very broadly and I’ll ask some more specific questions towards the very end of the interview.*

*We will be audio recording the interview, in order to capture all the details of our conversation. This is a purely voluntary activity, so remember that you may end the interview at any time. If you need to take a break, please let me know and we can do so. Also, if you feel uncomfortable with any question, tell me and we can skip it. No personal information about you will be shared with anyone outside of the study team. No identifying information will be shared in any reports or publications. While we might use direct quotes in such reports, they will only be attributed more generally to someone who is a “patient”, etc. All the information provided by you will be kept strictly confidential and we hope you will feel able to speak freely as we genuinely want to hear your perspective.*

*If a question does not make sense, let me know so I can ask it more clearly. Take as much time as you like to answer the questions.*

*Do you have any questions before we begin?*

*(and if not, start recording)….*

1. Our study is focused on people living with both diabetes and joint pain due to arthritis. I’m going to ask you about each of these conditions to learn more about how they impact your life. So first of all, tell me about your diabetes.

Probes:

- How long have you had diabetes?
- “How are you currently managing your diabetes – what is your current (treatment) approach?” Has this changed over time?”

1. So now, tell me about your joint pain. What has that experience been like?

Probes:

- How long have you had joint pain?
- Did a physician or other health care provider diagnose you with arthritis? If so, tell me about that experience.
  - When was that?
  - What did they tell you?
- Have you discussed your joint pain or arthritis diagnosis with the health care providers who treat your diabetes? What did *they* tell you?
- How active or troublesome do you feel your arthritis is right now?

1. What kinds of treatments have you tried to manage your joint pain?

Probes:

- Have you ever been seen by a physiotherapist or occupational therapist? If so, what was that experience like?
- Who suggested you see them (physiotherapist or occupational therapist)?
- Was this early on in your symptoms, or had they been going on for a while?
- Have you used any other treatments or therapies for your joint pain? Who prescribed or suggested it?

1. Our study is focused on people living with *both* diabetes and arthritis. So can you tell me what it is like to live with *both* diabetes *and* pain in your joints from arthritis?

Probes:

- How does it affect your everyday life? Your mood, being able to complete your daily tasks, keep up with friends and family, participate in activities that you enjoy?
- What kinds of challenges do you face living with both conditions?
- How does the one condition affect the other, in your view?

1. We’re interested in your experiences participating in physical activity (e.g. activities when you are moving around, like walking). Tell me about what kinds of physical activity you typically do. What does a typical day or week look like?

Probes:

- What activities do you like do? Have you been able to continuing doing those?
- Tell me about the activities you finder easier and also those you find more challenging to do?
- What seem to be the primary things that limit your ability to exercise or be active? (*Probes: are these symptoms related to having joint pain, or to diabetes?)*

1. How important is physical activity to you? How important is it for managing your diabetes? Your arthritis?

Probes:

- How satisfied are you with your current level of physical activity? What would you change if you could?
- There’s a lot of talk of physical activity these days. What role do you feel physical activity plays in the lives of people with diabetes? What about for people with arthritis?

1. Has a health care provider ever discussed physical activity with you? If so, tell me about that experience.

Probes:

- Who has discussed it with you?
- What did you find helpful?
- What did you find unhelpful?
- What would you like to know about physical activity?

1. What kind of supports, resources or tools do you think would help in keeping physically active OR allowing you to become more physically active?

Probes:

- If you had a new resource, what would that look like?
- What do you think health care providers could do to help you be more physically active or sustain your level of physical activity? Can you give me an example?
- What kind of information on physical activity would be helpful to you?
- How would you like to receive such information? From whom? In what formats?
- Have you used technology, such as wearable devices or apps, to help you be active in the past? What was your experience like?

1. Do you have anything else you wish to say related to your health, diabetes, joint pain or physical activity? Is there anything else you’d like to raise that we didn’t talk about today?
2. I would like to finish by asking about some demographic information and some details about your diabetes and arthritis:

What is your…

| Age | 40-49, 50-59, 60-69, 70+ |
| --- | --- |
| Gender | Male, Female, Other, Prefer not to answer |
| Location of residence | Urban, suburban, rural |
| Diabetes duration | 0-4 years, 5-9 years, 10-14 years, 15-20 years, 20-24 years, 25+ years |
| Diabetes treatment | No medications Oral medications Insulin (+/- oral meds) |
| Osteoarthritis duration | 0-4 years, 5-9 years, 10-14 years, 15-20 years, 20-24 years, 25+ years |
| HAQ mobility | Are you able to walk outdoors on flat ground?  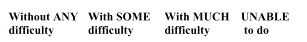  If difficulty or unable to: is this due to your arthritis? Yes/no |
| WOMAC pain | 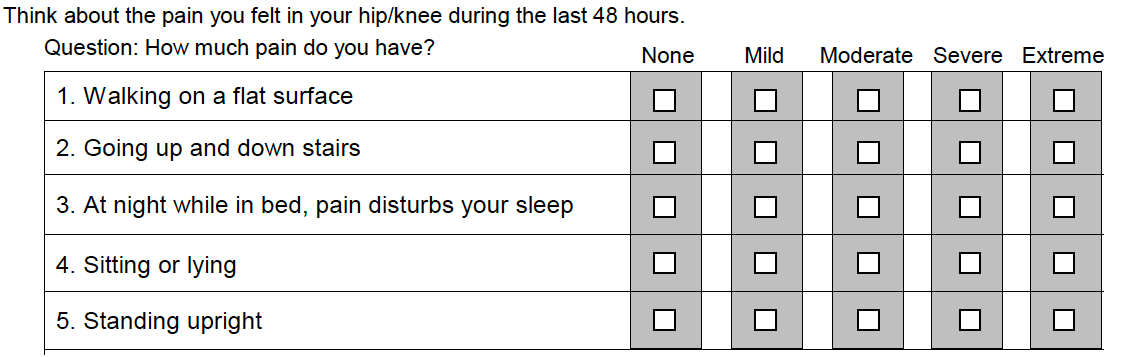 |

Thank you very much for your time and the information you shared today.

**Supplementary Table C**

**Interview Guide: Physicians and Diabetes Educators**

*Thank you for agreeing to speak with me today. We’re in the first phase of a study that will develop and test an intervention to improve treatment of joint pain in people with diabetes but before we start the trial we really want to hear what things are like currently at your institution and your perspectives.*

*I’m going to start the interview by asking you a few questions in order to get to know you and your practice a little bit more. Then I’m going to ask you about the perspective about physical activity in people with diabetes, and in particular your experiences of caring for patients with diabetes who might also have joint pain or arthritis.*

*I have a list of questions to go through that serve as a guide only. I encourage you to talk about any aspect of the topic you wish. There are no right or wrong answers to these questions.*

*I will be audio recording the interview, in order to capture all the details of our conversation. This is a purely voluntary activity, so remember that you may end the interview at any time. If you need to take a break, please let me know and we can do so. Also, if you feel uncomfortable with any question, tell me and we can skip it. No personal information about you will be shared and you will not be identified to your colleagues or patients. No identifying information will be shared in any reports or publications. While we might use direct quotes in such reports, they will only be attributed more generally to someone who is a “physician”, etc. All the information provided by you will be kept strictly confidential and we hope you will feel able to speak freely as we genuinely want to hear your perspective.*

*If a question does not make sense, let me know so I can ask it more clearly. Take as much time as you like to answer the questions.*

*Do you have any questions before we begin?*

*(and if not, start* *recording)….*

1. Can you start by describing your current practice?

Probes:

- What does your typical day look like?
- How long have you been in your current practice setting?
- Are there other clinicians in your practice? If so, what are their roles?
- Tell me a bit about the range and types of patients you see.
- How much of your practice involves diabetes care?

1. Tell me about your experiences seeing patients who have diabetes…just very generally, what are the things that you consider the priorities to cover in a routine follow up visit?

Probes:

- Typically, how long are the appointments?
- Recognizing that each clinical encounter is really busy, how to you decide what you can get to and what you can’t.

1. So the prevalence of multimorbidity is increasing. How do you approach the management of patients with diabetes who also have other chronic conditions – how do you juggle this? How do you think it affects your patients with diabetes?

Probes:

- Are some other conditions more top of mind, or given more consideration, than others? Which ones and why? Can you give me any examples?

1. When it comes to physical activity and being active, do you have a usual approach to talking about this with patients with diabetes? Can you give me some examples?

Probes:

- [M/A/D] Do you tend to bring up physical activity with all of your patients or do you wait for patients to initiate the discussion?
- [I] How top of mind is it? [BR] Do you have any tricks or tools you use to remind yourself to ask about physical activity in your patients with diabetes?
- [M/A/D] When someone is not active, does that prompt any further steps?
- [K] Do you ever refer to any guidelines or references? If so, which ones?

1. So, MSK problems and arthritis pain is very common in the general population. Does joint pain ever come up in your discussions with your diabetes patients?

Probes:

- How frequently do you have these kinds of conversations with your diabetes patients? What usually prompts the discussion?
- What kind of impact do you think joint pain has on their life? Their diabetes management?

1. Do your patients tend to seek health care with you or others specifically to manage their joint pains?

- How does assessment of their joint pain fit within your professional role or responsibility?
- How confident are you that you can assess a patient’s joint if they tell you they have joint pain? Tell me about a situation when you had to do a joint assessment?
- Tell me about any training you’ve received about musculoskeletal conditions, both diagnosing and treating them.
- Do you refer patients who report joint pain or arthritis to see any other health care practitioners? Who do you refer them to?
- Do you think addressing their arthritis will make a difference for your patients?

1. When it comes to helping patients with diabetes manage their joint pain, do you tend to recommend any specific therapies for them? What have you found helpful for these patients?

Probes:

- Are there any practitioners or programs that you refer to, to help with treatment? Does physiotherapy ever come up? What about other forms of therapy?
- Have you recommended any medications to manage pain?

1. I want to know about your thoughts on physical activity for patients living with *both* diabetes and joint pain or arthritis, especially in weight-bearing joints like the knee. How do you currently discuss physical activity with these patients? Do your recommendations change at all when they have both conditions? If so, how?

Probes:

- What informs your recommendations in the context of joint pain?
- Do you have any resources available to you that are helpful?
- Do you have any colleagues who are helpful when exploring physical activity with your patients?
- What do you see as the downsides of asking about and recommending physical activity to your diabetes patients who also have joint pain?

1. What kind of things, if any, would help you manage patients with both diabetes and joint pain or arthritis?

Probes:

- Are there any resources that would be helpful for you?
- What would help you make recommendations or referrals for physical activity for these patients? Please give me an example.
- Have you used toolkits or other reminder systems in the past? What was your experience like?

1. Is there anything else that you would like to comment on that I haven’t asked you about today?
2. I would like to finish with briefly asking a few demographic questions which we’re asking all our participants: What is your...

| Age | 20-29, 30-39, 40-49, 50-59, 60-69, 70+ |
| --- | --- |
| Gender | Man, Woman, Other, Prefer not to answer |
| Professional designation | Family physician, endocrinologist, diabetes educator |
| Number of years in practice | 0-4, 5-9, 10-14, 15-19, 20-24, 25-29, 30+ |
| Location of practice | Urban, suburban, rural |
| Is University affiliated practice? | Yes, no |

Thank you very much for your time and the information you shared today.

**Supplementary Table D**

**Interview Guide: Arthritis Therapists**

*Thank you for agreeing to speak with me today. I’m just going to give you a bit of an overview of what to expect from this interview. I’m going to begin by asking you a few questions in order to get to know you and a little bit about your role at the Arthritis Society. Then I’m going to ask you about your perspectives on knee osteoarthritis in people with diabetes, and, in particular, your experiences with assessing and providing recommendations for patients with knee osteoarthritis who also have diabetes. We are planning a clinical trial to test an intervention designed to improve the treatment of joint pain in people with diabetes. Before we start that study, we really want to hear about your current practices with patients who have both of these conditions to help inform the intervention for that trial. These questions serve as a guide only. We encourage you to talk about any aspect of the topic you wish. There are no right or wrong answers to these questions..it’s just your experience and perceptions.*

*We will be audio recording the interview, in order to capture all the details of our conversation. This is a purely voluntary activity, so remember that you may end the interview at any time. If you need to take a break, please let me know and we can do so. Also, if you feel uncomfortable with any question, tell me and we can skip it. No personal information about you will be shared with anyone outside of the study team and you will not be identified to your colleagues or patients. No identifying information will be shared in any reports or publications. While we might use direct quotes in such reports, they will only be attributed more generally to someone who is a “arthritis therapist”, etc. All the information provided by you will be kept strictly confidential and we hope you will feel able to speak freely as we genuinely want to hear your perspective.*

*If a question does not make sense, let me know so I can ask it more clearly. Take as much time as you like to answer the questions.*

*Do you have any questions before we begin?*

*START RECORDER.*

STATE: Name of therapist, date and time of interview.

1. Let’s start with some background about you. What can you tell me about your clinical training?

Probes:

- Do you have any specific training about DM?
- Tell me about your experiences and level of comfort caring for patients who have other chronic conditions other than arthritis.

1. Now, let’s turn to your clinical practice. Can you tell me about your current practice as an arthritis therapist?

Probes:

- How long have you been in practice? How long, specifically, as an arthritis therapist?
- Approximately, what % of your caseload would consist of OA patients…how many of those have both OA and DM?
- Describe a “typical” OA patient that you would see.

1. So I’m sure many of the patients you see also have other things going on with their health. Do you routinely ask about other health problems? How do patients’ other conditions, if present, factor into your treatment plan?
2. Let’s talk more about your patients with both OA and DM. Describe how you typically manage a patient with both OA and DM. Can you give an example of what management might look like for a typical patient?

Probes: (General management)

- What types of issues tend to come up with these patients?
- Do you manage an OA patient differently if they have DM? If so, in what ways?
- How long are they under your care?
- Where might you refer them after discharge?

Probes: (Re. Physical Activity)

- What types of PA do you typically recommend to these patients? Is this typically therapeutic strengthening exercises or aerobic exercise, or both?
- Do you focus at all on increasing their general level of PA? (or is the focus on therapeutic exs)
- How does having DM alter your approach to prescribing PA?
- What informs your PA recommendations? (e.g. guidelines, other evidence)

1. Do you face any challenges with respect to prescribing PA for patients with both OA and DM? How do you address these challenges?

Probes:

- What do you feel are the main barriers to adherence to PA among the patients you see?
- What strategies do you use to support and motivate patients to participate in physical activity?
- What strategies do you think work well to get patients to increase their level of physical activity? What kind of strategies do you find helpful for patients to sustain an increase in PA?

1. Are you familiar with the concept of behavioural change techniques?? Tell me about your experience with behavioural change techniques such as coaching or motivational interviewing to support PA with your patients.

Probes:

- What support(s) would you need to be able to incorporate (or, if currently using: optimize use of) behavioural change strategies into your practice (e.g. goals setting, actional planning)?

1. Is there anything you need to help you further in supporting patients with OA and DM to participate in physical activity?

Probes:

- What kinds of support or information related to DM do you need to help manage these patients?

1. Is there anything else that you would like to comment on that I haven’t asked you about today?
2. I would like to finish by asking about some demographic information:

What is your…

| Age | 20-29, 30-39, 40-49, 50-59, 60-69, 70+ |
| --- | --- |
| Gender | Male, Female, Other, Prefer not to answer |
| Professional designation | Physical therapist, Occupational therapist |
| Number of years in practice | 0-4, 5-9, 10-14, 15-19, 20-24, 25-29, 30+ |
| Location of practice | Urban, suburban, rural |

Thank you very much for your time and the information you shared today.

STATE: This is the End of the recording.
